# Supplementary material for: Malonylation of GAPDH is an inflammatory signal in macrophages
Source: Nat Commun. 2019 Jan 18;10:338. doi: 10.1038/s41467-018-08187-6 (PMC6338787; doi:10.1038/s41467-018-08187-6)

Supplementary information

Malonylation of GAPDH is an inflammatory signal in macrophages

Galván-Peña *et al*

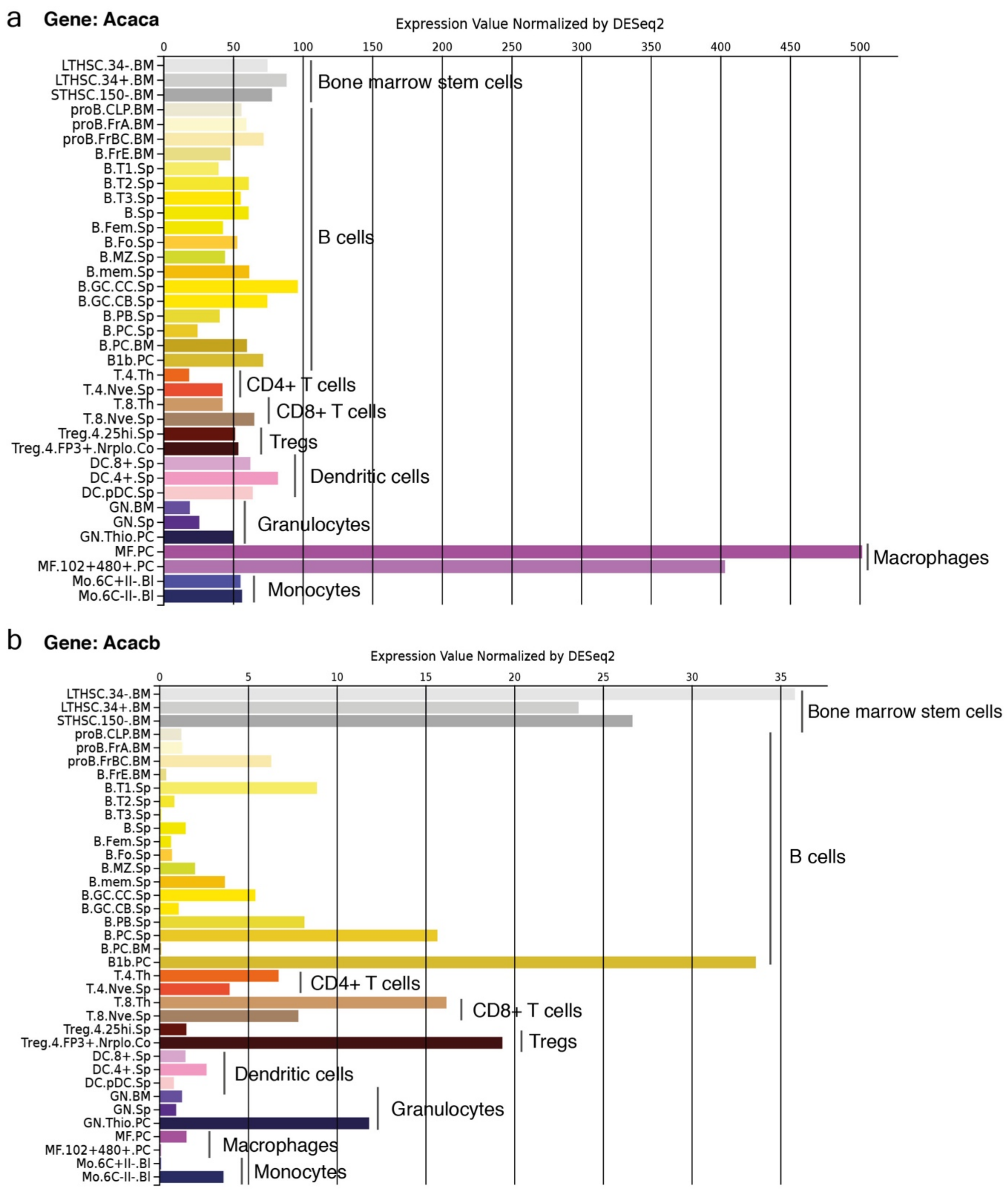

a
Gene: Acaca
Expression Value Normalized by DESeq2
0
50
100
150
200
250
300
350
400
450
500
LTHSC.34-.BM
LTHSC.34+.BM
STHSC.150-.BM
proB.CLP.BM
proB.FrA.BM
proB.FrBC.BM
B.FrE.BM
B.T1.Sp
B.T2.Sp
B.T3.Sp
B.Sp
B.Fem.Sp
B.Fo.Sp
B.MZ.Sp
B.mem.Sp
B.GC.CC.Sp
B.GC.CB.Sp
B.PB.Sp
B.PC.Sp
B.PC.BM
B1b.PC
T.4.Th
T.4.Nve.Sp
T.8.Th
T.8.Nve.Sp
Treg.4.25hi.Sp
Treg.4.FP3+.Nrplo.Co
DC.8+.Sp
DC.4+.Sp
DC.pDC.Sp
GN.BM
GN.Sp
GN.Thio.PC
MF.PC
MF.102+480+.PC
Mo.6C+II-.Bl
Mo.6C-II-.Bl
Bone marrow stem cells
B cells
CD4+ T cells
CD8+ T cells
Tregs
Dendritic cells
Granulocytes
Macrophages
Monocytes
b
Gene: Acacb
Expression Value Normalized by DESeq2
0
5
10
15
20
25
30
35
LTHSC.34-.BM
LTHSC.34+.BM
STHSC.150-.BM
proB.CLP.BM
proB.FrA.BM
proB.FrBC.BM
B.FrE.BM
B.T1.Sp
B.T2.Sp
B.T3.Sp
B.Sp
B.Fem.Sp
B.Fo.Sp
B.MZ.Sp
B.mem.Sp
B.GC.CC.Sp
B.GC.CB.Sp
B.PB.Sp
B.PC.Sp
B.PC.BM
B1b.PC
T.4.Th
T.4.Nve.Sp
T.8.Th
T.8.Nve.Sp
Treg.4.25hi.Sp
Treg.4.FP3+.Nrplo.Co
DC.8+.Sp
DC.4+.Sp
DC.pDC.Sp
GN.BM
GN.Sp
GN.Thio.PC
MF.PC
MF.102+480+.PC
Mo.6C+II-.Bl
Mo.6C-II-.Bl
Bone marrow stem cells
B cells
CD4+ T cells
CD8+ T cells
Tregs
Dendritic cells
Granulocytes
Macrophages
Monocytes

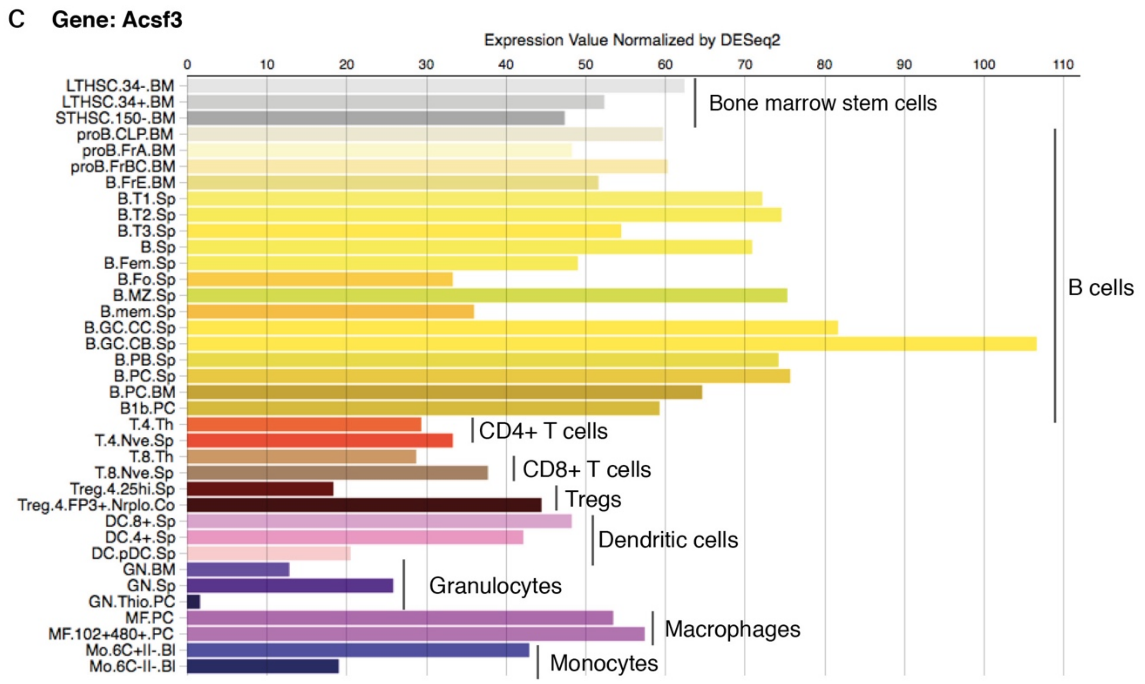

Supplementary figure 1. ACC1, ACC2 and ACSF3 gene expression across immune cell types.

Acaca, *Acacb* and *Acsf3* gene expression data from ULI RNASeq, obtained using the RNASeq Skyline data browser from the Immgen Consortium. BM (bone marrow); Sp (spleen); PC (peritoneal cavity); Co (colon); Bl (blood); LTHSC (long term hematopoietic stem cells); STHSC (short term hematopoietic stem cells); CLP (common lymphoid progenitor); Fo (follicular); MZ (marginal zone); mem (memory); fem (female); GC.CC (germinal centres centrocytes); GC.CB (germinal centres centroblasts); PB (plasmablasts); T4.Th (CD4+ thymocytes); T8.Th (CD8+ thymocytes); GN (neutrophil); MF (macrophage).

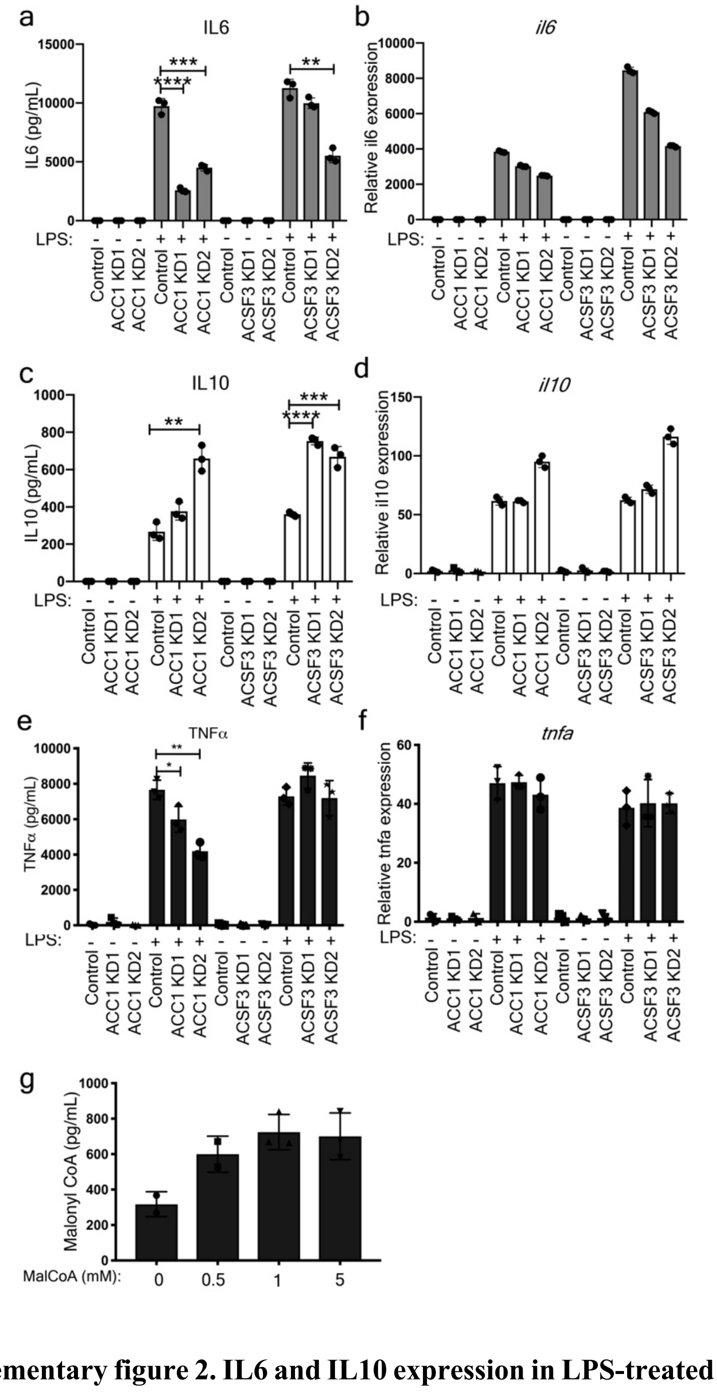

Supplementary figure 2. IL6 and IL10 expression in LPS-treated ACC1 and ACSF3 KD BMDMs.

a, IL6 protein measured by ELISA (24h, mean + SEM, n=3) and **b,** IL6 mRNA expression in 100 ng/mL LPS-treated ACC1 and ACSF3 KD BMDMs (6h, mean + SD, representative of three independent experiments). **c**, IL10 protein measured by ELISA (24h, mean +SEM, n=3) and **d**, IL10 mRNA expression in 100 ng/mL LPS-treated ACC1 and ACSF3 KD BMDMs, (24h, mean +SD, representative of three independent experiments). **e,** TNFα protein measured by ELISA (mean + SEM, n=3) and **f,** TNFα mRNA expression in 100 ng/mL LPS-treated (100 ng/mL, 24h) ACC1 and ACSF3 KD BMDMs (mean + SD, representative of three independent experiments) **g,** Malonyl-CoA levels measured in malonyl-CoA treated BMDMs using a malonyl-CoA ELISA. Unpaired student's t test, **$p < 0.01$; *** $p < 0.005$; **** $p < 0.001$.

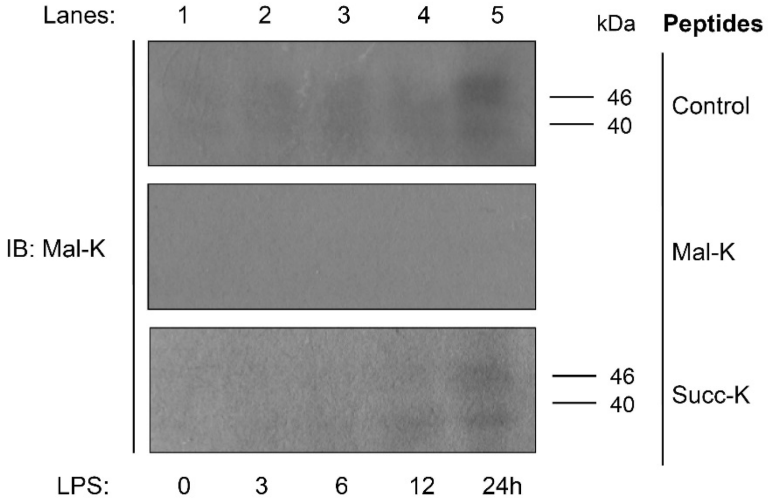

Supplementary figure 3. Testing of anti-malonyl-lysine antibody specificity through peptide competition.

Anti-mal-K antibody was pre-incubated with either succ-K peptide, mal-K peptide, or no peptide, prior to its use in the analysis of mal-K levels via western blotting. BMDMs were treated with 100 ng/mL LPS. Results representative of three independent experiments.

a

MS/MS spectrum database search analysis summary			
Identified proteins	Quantifiable proteins	Identified sites	Quantifiable sites
513	412	1075	843

b

Sites differentially modified by LPS		
Regulation	Proteins	Sites
Upregulated	98	123
Downregulated	12	13

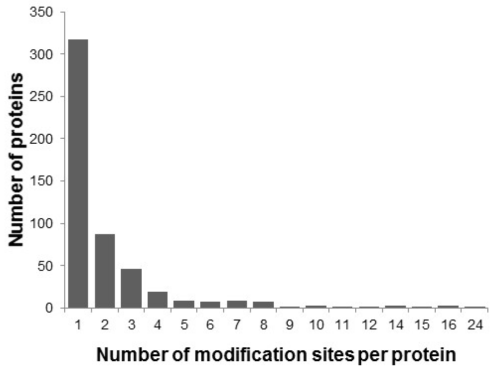

c

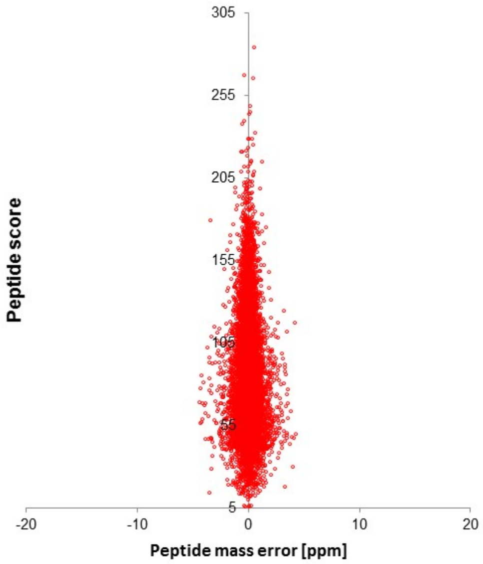

Supplementary figure 4. Malonylated peptides identified on untreated and LPS-treated BMDMs

a, Summary of the results obtained from the identification of malonylated peptides in bone marrow-derived macrophages through mass spectrometry. **b,** Number of statistically significant proteins and sites identified as undergoing malonylation (≥ 1.5 fold) following 24h of LPS treatment (p value < 0.05), or present only in untreated vs LPS-treated samples (n=3). **c,** Peptide scores and peptide mass error measured for quality control of the peptides identified via mass spectrometry.

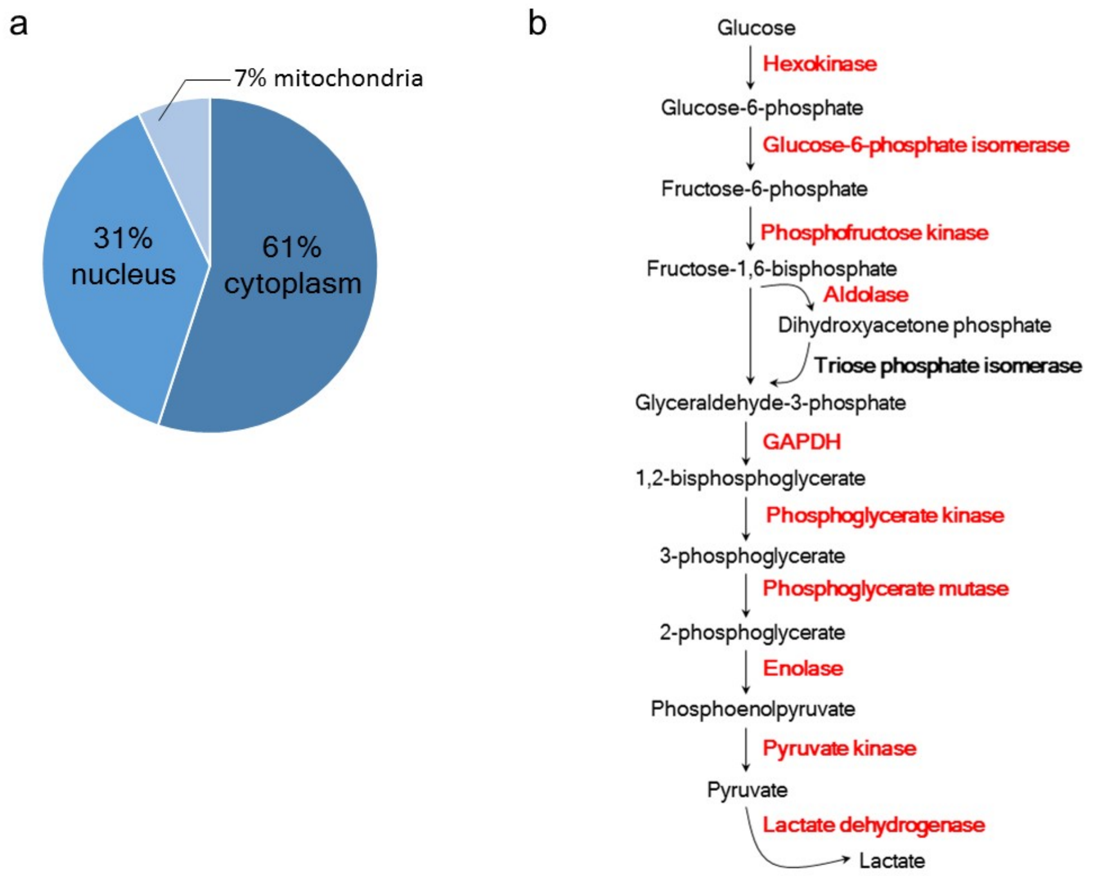

Supplementary figure 5. a, Cellular distribution of identified LPS-malonylated proteins. **b,** LPS-malonylated enzymes in glycolysis, as identified via mass-spectrometry.

GAAQNIIPASTGAAKmalAVGK

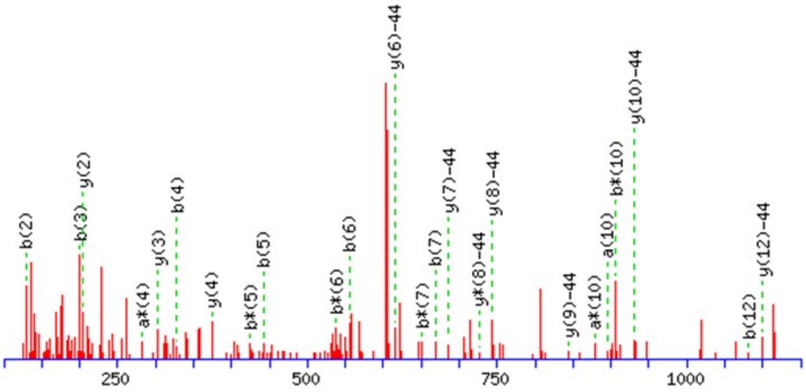

TVDGPSGKmalLWR

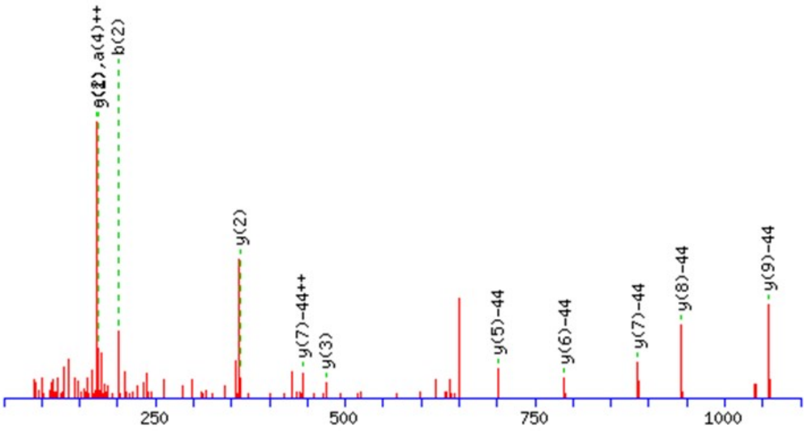

Supplementary figure 6. MS/MS spectra of malonylated peptides identified in GAPDH

MS/MS spectra from the malonylated peptides detected on a Q-Exactive Orbitrap mass spectrometer following immunoprecipitationg of GAPDH from BMDMs and trypsin-digestion.

a

```
                                   |K213
Human          KLWRDGRGAAQNIIPASTGAAKAVGKVIPELNGKLTGMAFRVPTPNVSVV 241
Mouse          KLWRDGRGAAQNIIPASTGAAKAVGKVIPELNGKLTGMAFRVPTPNVSVV 241
Rat            KLWRDGRGALQNIIPASTGAAKAVGKVIPELDGKLTGMAFRVPTANVSVV 243
C. Elegans     KLWRDGRGAGQNIIPASTGAAKAVGKVIPELNGKLTGMAFRVPTPDVSVV 249
E.Coli         KDLRASRAAAENIIPHTTGAAKAIGLVIPELSGKLKGHAQRVPVKTGSVT 241
```

b

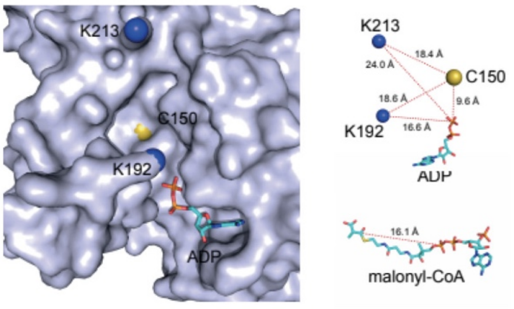

c

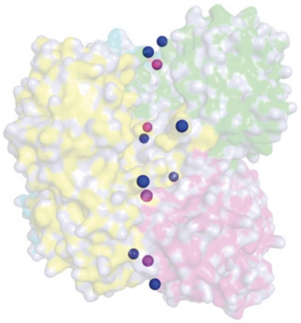

Supplementary figure 7. K213 sequence alignment and positioning in the GAPDH structure.

a, Sequence alignment of an amino acid fragment of GAPDH across multiple species. **b,** The ADP moiety of NAD is shown in the NAD-bound structure of bovine GAPDH (4O59), as well as the geometry of the ADP-binding site relative to C150, K192 and K213, together with the flexible pantetheine moiety of malonyl-CoA. **c**, K192, K213 (blue) and T227 (pink) positioning along the dimerization and RNA-binding cleft of GAPDH.

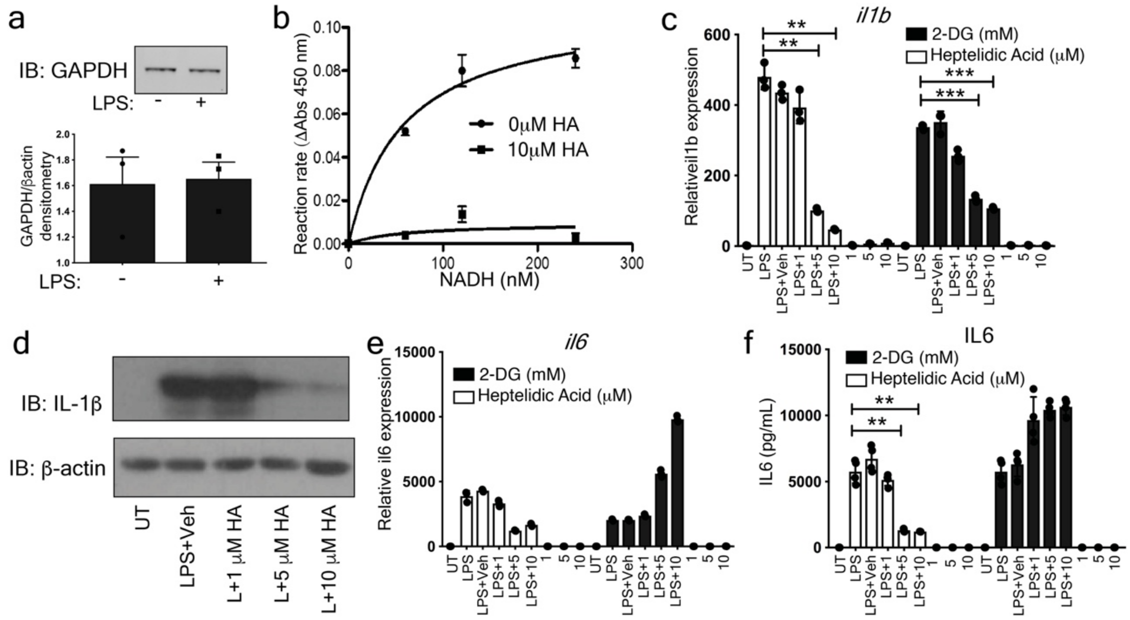

Supplementary figure 8. HA inhibits GAPDH enzymatic activity and also blocks IL1β and IL6 production.

a, GAPDH expression in untreated and LPS-treated (24h) BMDMs **b,** GAPDH enzymatic activity measured at 340 nm with various concentrations of NADH in lysates treated with 10 μM HA**. c**, IL1β mRNA expression in 100 ng/mL LPS-treated BMDMs (6h), pre-treated with HA or 2-DG (mean + SEM, n=4). **d**, Pro-IL1β protein measured by western blotting in 100 ng/mL LPS-treated BMDMs pre-treated with HA. **e**, IL6 mRNA expression and **f**, IL6 protein expression in 100 ng/mL LPS-treated BMDMs (6h), pre-treated with HA or 2DG (mean + SEM, n=4). Unpaired student's t test, $^{**}p < 0.01$; $^{***}p < 0.005$

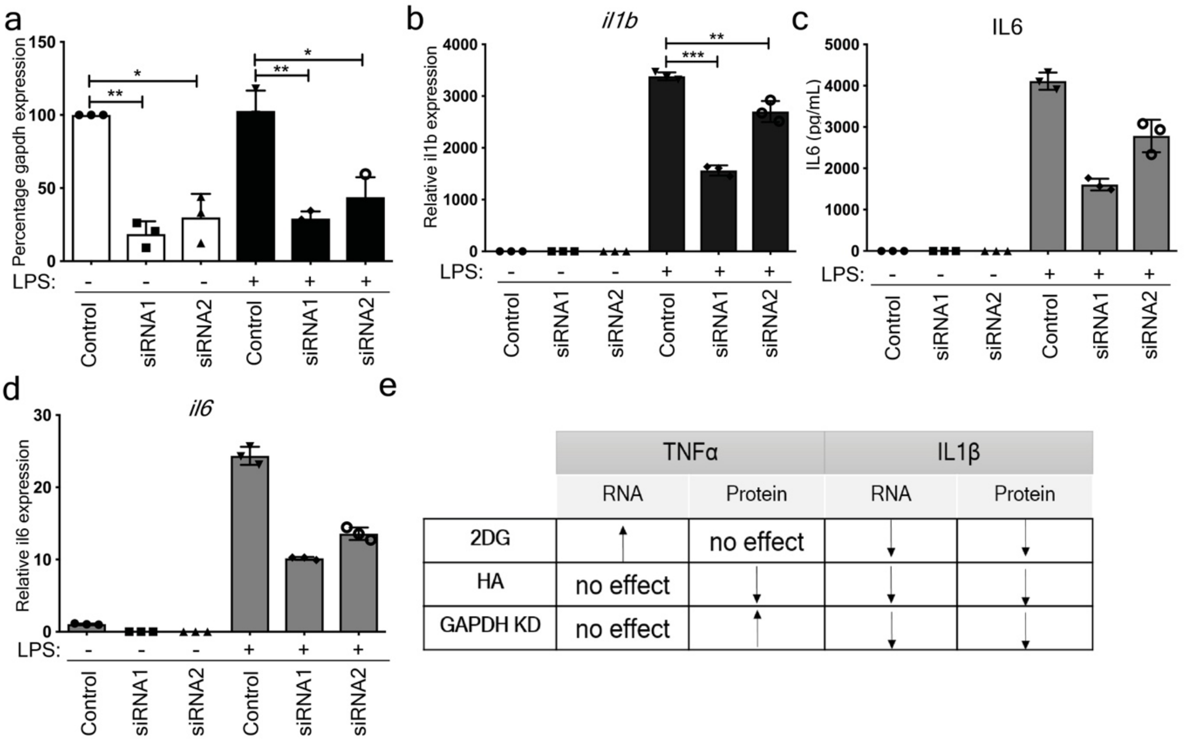

Supplementary figure 9. GAPDH KD reduces IL1β and IL6 production

a, GAPDH expression following siRNA KD (72h, 10nM), measured by qPCR and normalised to 18S expression, and depicted as percentage relative to control (mean + SEM,n=3) **b**, IL1β mRNA expression in 100 ng/mL LPS-treated GAPDH KD BMDMs. **c**, IL6 protein measured by MSD and **d**, IL6 mRNA expression measured by qPCR in LPS-treated GAPDH KD BMDMs. **e**, Summary of HA, 2DG and GAPDH KD results. Unpaired student's t test, $*p < 0.05$; $**p < 0.01$; $***\ p < 0.005$.

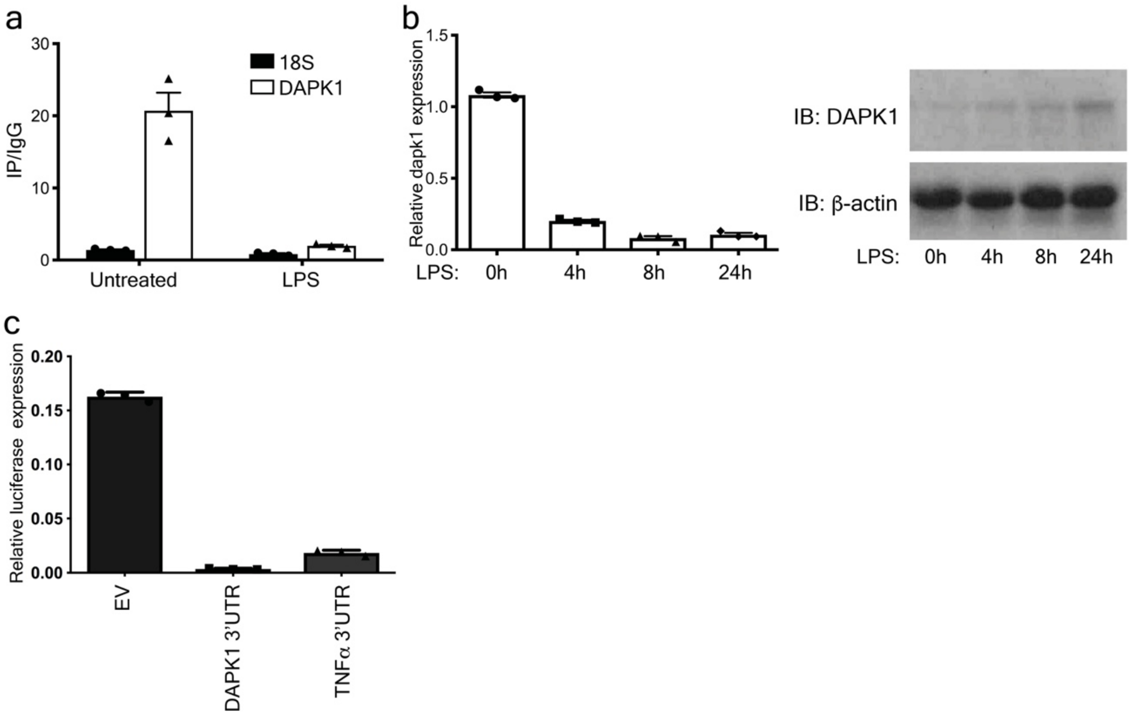

Supplementary figure 10. GAPDH negatively regulates DAPK1 through RNA-binding

a, GAPDH was immunoprecipitated from untreated and LPS-treated (24h, 100 ng/mL) BMDMs, and DAPK1 mRNA presence assessed via qPCR. **b,** DAPK1 mRNA expression measured by qPCR and DAPK1 protein measured via western blotting in BMDMs treated with LPS (100 ng/mL) over time. **c,** Luciferase activity of an empty vector (EV), DAPK1 3'-UTR, TNFα 3'UTR luciferase constructs. Renilla luciferase expression was normalised to firefly luciferase activity. Mean + SD of three independent experiments.

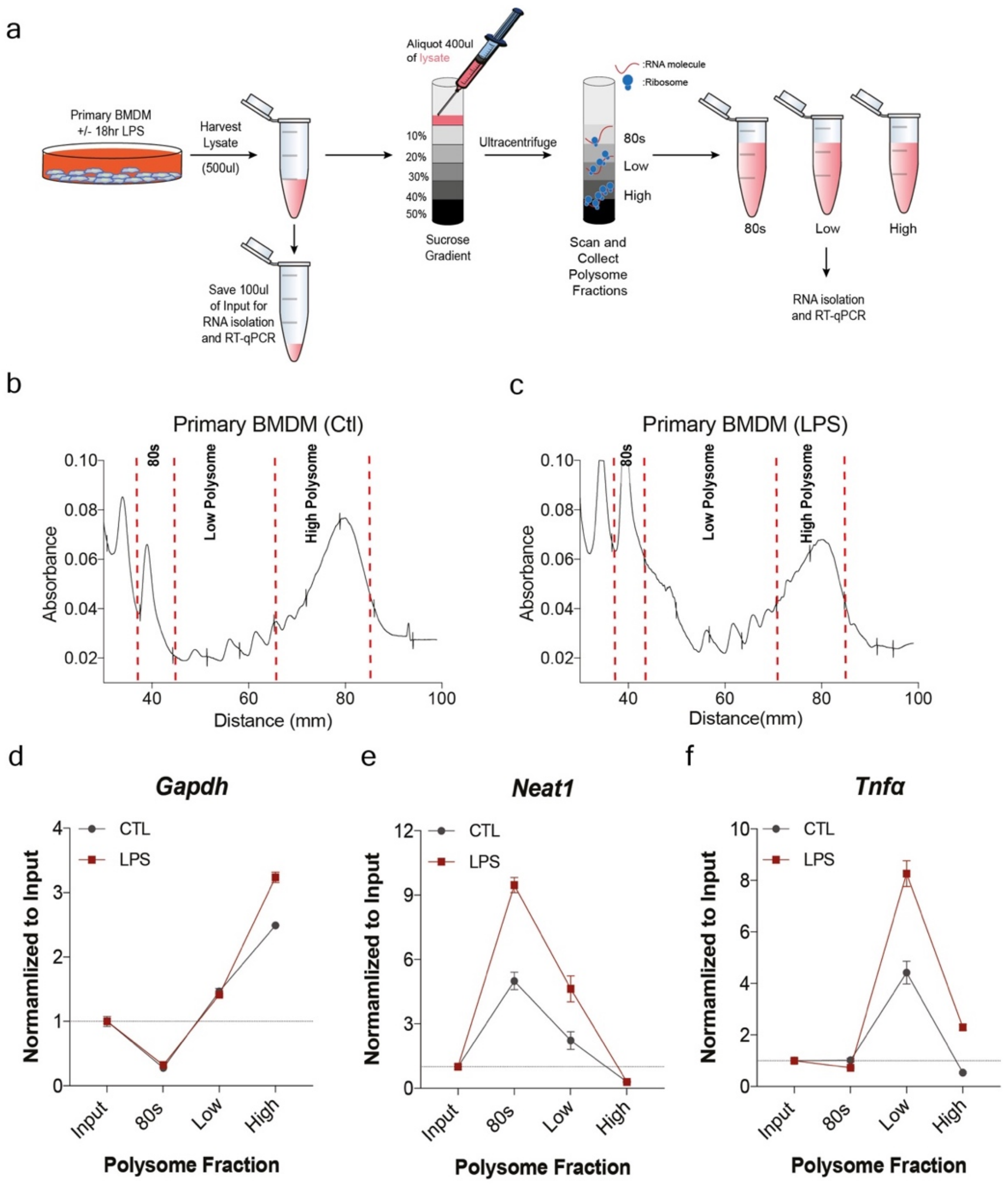

Supplementary figure 11. LPS increases TNFα translation

a, Overview of the polysome profiling protocol to analyze translation activity. The various steps of the protocol involve cell lysis, sucrose gradient centrifuge, RNA extraction and analysis of translation status of mRNAs by RT-qPCR. **b, c,** Cytoplasmic lysates from control (b) and LPS treated cells (c) were fractionated through sucrose gradients. Global RNA polysome profiles generated by the density gradient fractionation system are shown. Results representative of four independent experiments. **d,e,f,** The relative distribution of *Gapdh* mRNA (d), encoding a housekeeping protein, *Neat1* long non-coding RNA (lncRNA) (e) and *Tnfα* mRNA (f) were measured by RT-qPCR analysis of RNA. Each of the gradient fractions are calculated as relative enrichment when compared to unfractionated input mRNA. Experiment performed in biological quadruplicates.

a

Protein	Ribosomal subunit	Fold induction
Rpl7a	60S	LPS only
Rpl10a	60S	LPS only
Rpl13a	60S	LPS only
Rpl7	60S	LPS only
Rps4x	40S	LPS only
Rps3	40S	LPS only
Rps2	40S	LPS only
Eef2	Elongation factor	LPS only
Rpl11	60S	LPS only
Rpl26	60S	LPS only
Rpl4	60S	13.6
Rpl18a	60S	8.2
Rpl6	60S	7.8
Rpl8	60S	3.3
Rps8	60S	2.98
Rpl18a	60S	2.9
Rps9	40S	2.6
Eef1a	Elongation factor	2.3

b

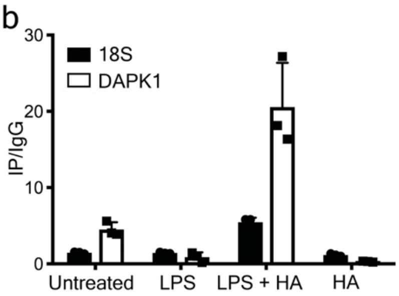

Supplementary figure 12. GAPDH-interacting translation-associated proteins and HA effect on DAPK1 RNA-binding

a, Proteins associated with translation identified as interacting with GAPDH via mass spectrometry in BMDMs treated with LPS (100ng/mL, 24h) when compared to untreated BMDMs (minimum 2-fold). **b**, LPS-treated (100 ng/mL, 24h) BMDMs pre-treated with HA (10 µM) and GAPDH immunoprecipitated. Bound DAPK1 mRNA was assessed by qPCR. Data shown is representative of three independent experiments.

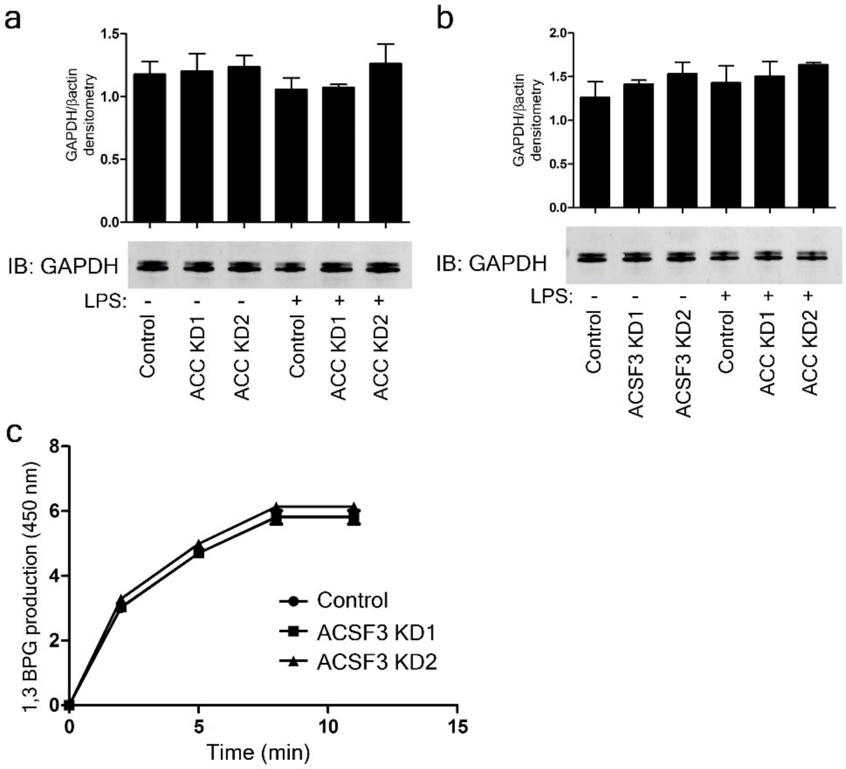

Supplementary figure 13. GAPDH expression and enzymatic activity in ACC1 and ACSF3 KD.

GAPDH expression in **a**, ACC1 and **b**, ACSF3 KD BMDMs. **c,** GAPDH enzymatic activity in ACSF3 KD (LPS-treated, 100 ng/mL, 24h).

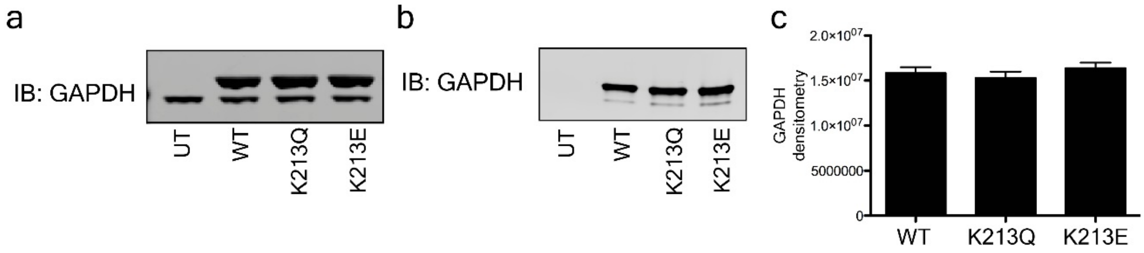

Supplementary figure 14. Expression of WT, K213Q and K213E GAPDH mutants.

a, GAPDH expression in HEK293T transfected with WT, K213Q and K213E constructs. **b,** Expression levels of affinity-purified GAPDH variants. **c,** Quantification of GAPDH affinity-purified variants from western blot expression using the image studio software. Mean + SEM of three independet blots.

Supplementary figure 15 – uncropped western blot images from the main figures

Figure 1c

ACC1c

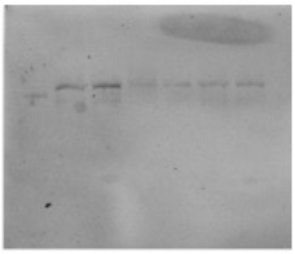

β-actin

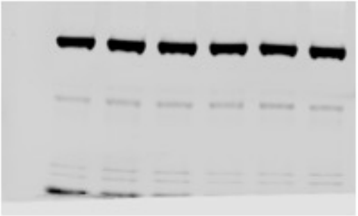

Figure 2a

Mal-K

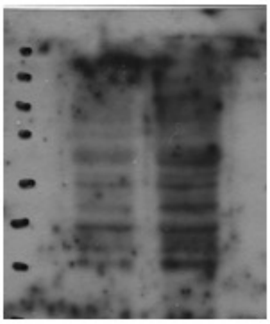

β-actin

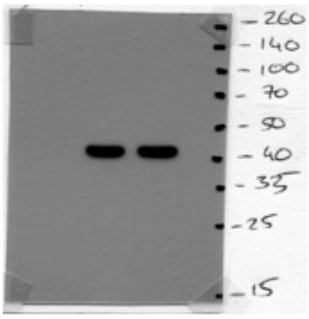

Figure 2c

GAPDH

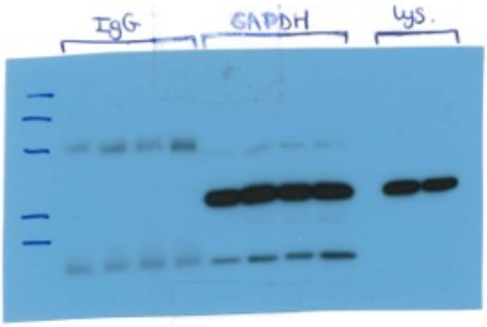

Mal-K

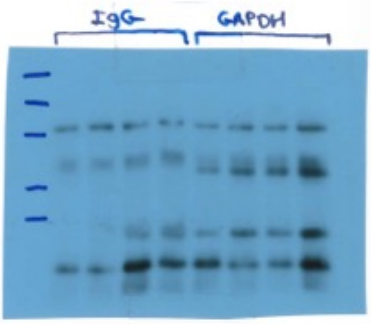

Figure 2d

GAPDH

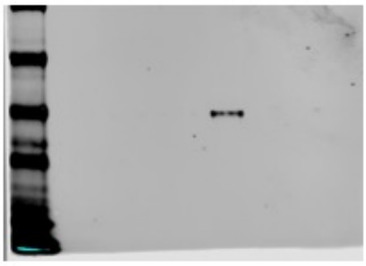

Figure 2f

GAPDH

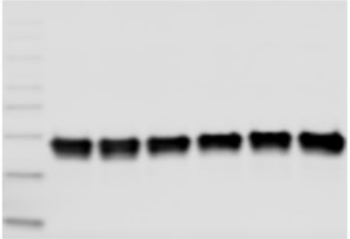

Mal-K

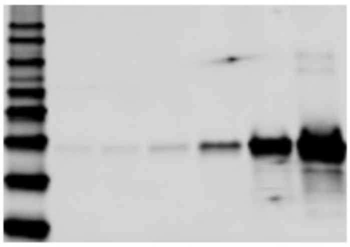

Figure 2h

GAPDH

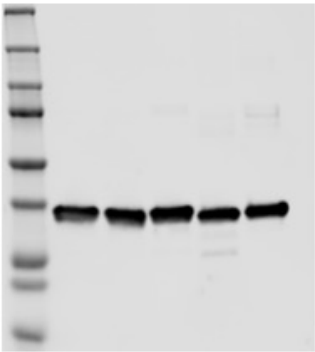

Mal-K

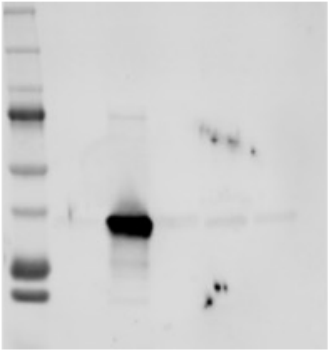

Figure 3d

GAPDH

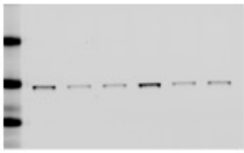

IL1-β

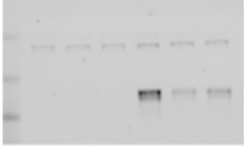

β-actin

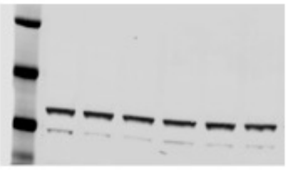

Figure 4d

GAPDH

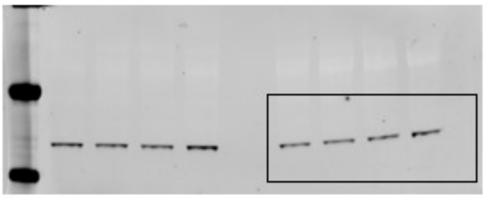

β-actin

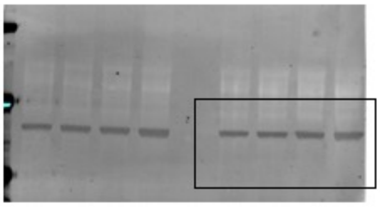

Figure 5c

GAPDH

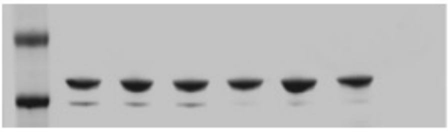

myc

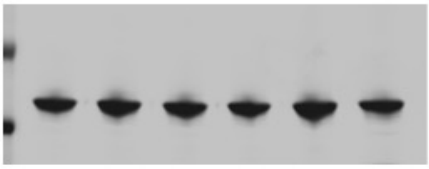

Supplement: Supplementary file 1 — Supplementary information [file 41467_2018_8187_MOESM1_ESM.pdf]
