# Supplementary material for: Malonylation of GAPDH is an inflammatory signal in macrophages
Source: Nat Commun. 2019 Jan 18;10:338. doi: 10.1038/s41467-018-08187-6 (PMC6338787; doi:10.1038/s41467-018-08187-6)
Supplement: Supplementary file 3 — Description of Additional Supplementary Files [file 41467_2018_8187_MOESM3_ESM.docx]

**Description of Additional Supplementary Files**

**File Name:** Supplementary data 1

**Description:** Identification of malonylated peptides in lysates from BMDMs untreated or treated with LPS for 24h (100 ng/mL). Trypsin-digested peptides were analysed via LC-MS (n=3).

**File Name**: Supplementary Data 2

**Description**: Post-translational modifications identified in GAPDH immunoprecipitated from BMDMs untreated or treated with LPS for 24h (100 ng/mL). Trypsin-digested peptides were analysed via LC-MS (n=3).

**File Name:** Supplementary Data 3

**Description:** Proteins identified as interacting with GAPDH co-immunoprecipitated from BMDMs untreated or treated with LPS for 24h (100 ng/mL). Trypsin-digested peptides were analysed via LC-MS (n=3).
